# Integrated Metabolomic and Lipidomic Analysis in the Placenta of Preeclampsia

**DOI:** 10.3389/fphys.2022.807583

**Published:** 2022-02-04

**Authors:** Lizi Zhang, Shilei Bi, Yingyu Liang, Lijun Huang, Yulian Li, Minshan Huang, Baoying Huang, Weinan Deng, Jingying Liang, Shifeng Gu, Jingsi Chen, Lili Du, Dunjin Chen, Zhijian Wang

**Affiliations:** ^1^Department of Obstetrics and Gynecology, Nanfang Hospital, Southern Medical University, Guangzhou, China; ^2^Department of Obstetrics and Gynecology, Key Laboratory for Major Obstetric Diseases of Guangdong Province, The Third Affiliated Hospital of Guangzhou Medical University, Guangzhou, China; ^3^Guangdong-Hong Kong-Macao Greater Bay Area Higher Education Joint Laboratory of Maternal-Fetal Medicine, Guangzhou, China; ^4^Guangdong Engineering and Technology Research Center of Maternal-Fetal Medicine, Guangzhou, China

**Keywords:** preeclampsia, metabolomics, lipidomics, ultra-high performance chromatography mass spectrometry, WGCNA

## Abstract

Preeclampsia is one of the most common severe pregnancy complications in obstetrics, which is considered a placental source disease. However, the mechanisms underlying preeclampsia remain largely unknown. In this study, UPLC-MS/MS-based metabolomic and lipidomic analysis was used to explore the characteristic placental metabolites in preeclampsia. The results revealed that there were significant changes in metabolites between preeclampsia and normotensive placentas. Weighted correlation network analysis (WGCNA) identified the correlation network module of metabolites highly related to preeclampsia and the clinical traits reflecting disease severity. The metabolic perturbations were primarily associated with glycerophospholipid and glutathione metabolism, which might influent membrane structures of organisms and mitochondria function. Using linear models, three metabolites had an area under receiver operating characteristic curves (AUROC) ≥ 0.80 and three lipids had an AUROC ≥ 0.90. Therefore, metabolomics and lipidomics may offer a novel insight for a better understanding of preeclampsia and provide a useful molecular mechanism underlying preeclampsia.

## Introduction

Preeclampsia is one of the most common severe pregnancy complications in obstetrics, with new-onset hypertension and other organs dysfunction and accompanied by new-onset proteinuria or not (ACOG Practice Bulletin No.202, 2019). The incidence of preeclampsia is 2–8% (ACOG Practice Bulletin No.222, 2020) and causes about 70,000 maternal and 500,000 fetal deaths globally every year (Rana et al., 2019). Furthermore, the effects of preeclampsia on substantial cardiovascular disease and cerebrovascular disease of women and fetuses are life-long (Bergman et al., 2020; Hoodbhoy et al., 2021). Though some researchers have provided important insights into the pathogenesis of preeclampsia and its related complications over the last decade (Karumanchi and Granger, 2016), the mechanisms leading to preeclampsia are only partially known.

It is considered that preeclampsia is a placental disease. Placentas from women with preeclampsia display an increased frequency of infarcts of villous, villous-free placental lakes, inflammation, fibrin deposition, syncytial knots, and abnormal cytotrophoblast proliferation (Granger et al., 2001; Falco et al., 2017; Burton et al., 2019). It is commonly accepted that the pathophysiology of preeclampsia is uteroplacental mal-perfusion secondary to defective remodeling of the uterine spiral arteries by a failure of trophoblast invasion (Granger et al., 2001). Preeclampsia is also associated with changes in gene expression (Zheng et al., 2020) and placental DNA methylation (Wei et al., 2020). Factors released from the placenta, including exosomes, pro-inflammatory cytokines, cell-free fetal DNA, and anti-angiogenic agents into systemic circulation disrupt maternal endothelial function, which results in the clinical multisystem syndrome of preeclampsia (Tannetta et al., 2017). However, up to now, except for timely labor induction and pregnancy termination, there is no effective predictive tool and treatment for preeclampsia. The changes of the placenta and the mechanism of preeclampsia remain to require further elucidation.

In contrast to genomics, transcriptome and proteomics, metabolomics, a systematic approach that studies the number and variety of endogenous metabolites caused by the pathophysiological changes of external stimuli and mutations of the organism itself, can more directly and accurately reflect the state of the organism (Rinschen et al., 2019). Metabolites are downstream products of the genome and proteome, which are highly relevant to the phenotype and function in this dynamic system (Kawasaki et al., 2019; Rinschen et al., 2019). In addition, lipidomics, a subcategory of metabolic profiling, can efficiently analyze lipid family and lipid molecule changes in various pathophysiological processes. Lipids are the main components of membrane structures of organisms (such as outer membrane, mitochondria, endoplasmic reticulum, and exosomes), as well as signal small molecules and energy substances (Avela and Siren, 2020). The disturbance of lipid metabolism is closely related to the occurrence and development of various diseases, such as cardiovascular metabolic syndrome (Soppert et al., 2020), tumors (Butler et al., 2020), and neurodegenerative diseases (Kao et al., 2020). Quantitative analysis of metabolites may help to characterize the metabolic perturbations, and metabolites that are markedly altered in pathological conditions can be used as possible clinical markers for diagnosis and treatment.

Studying the metabolic perturbations of the placenta in preeclampsia, and how it is affected by disease characteristics, may give insight into which pathological processes are specific for these disorders. In this study, ultra-high performance liquid chromatography-mass spectrometry (UPLC-MS) was applied to investigate metabolomics and lipidomics profiling of placentas from preeclampsia and normotensive pregnant women. Therefore, metabolomics and lipidomics may offer a novel insight for a better understanding of preeclampsia and provide a useful molecular mechanism underlying preeclampsia.

## Materials and Methods

### Subjects and Placenta Tissue

This case-control study and placenta collection were approved by the Medical Ethics Committee of The Third Affiliated Hospital of Guangzhou Medical University with Medical Research No. 2018022.

Preeclampsia was defined as new-onset hypertension after 20 weeks of gestation with systolic blood pressure ≥ 140 mm Hg or diastolic blood pressure ≥ 90 mm Hg on two occasions at least 4 h apart after, and with proteinuria ≥ 300 mg per 24 h urine or dipstick reading of 2+ (used only if quantitative methods not available), or other organ dysfunction in the absence of proteinuria (ACOG Practice Bulletin No.202, 2019). Preeclampsia is subdivided into early and late onset phenotypes, diagnosed before 34 weeks or from 34 weeks of gestation, respectively.

This study included 12 preeclampsia pregnant women and 14 normotensive pregnant women who delivered by cesarean section in the Department of Obstetrics and Gynecology, Third Affiliated Hospital of Guangzhou Medical University. We excluded pregnant women complicated with diabetes mellitus, metabolic syndrome, twin pregnancy, complicated with hepatitis B virus infection, and syphilis infection. The detailed patient characteristics are presented in Table 1. The tissue samples were obtained from the central part of the fetal placenta avoiding macroscopic vessels and areas of calcification and infarction immediately after delivery of the placentas. After rinsing in saline, the samples were snap-frozen in liquid nitrogen and stored at −80°C for metabolomics and lipidomics analysis.

**TABLE 1:** The characteristics of the preeclampsia and normotensive pregnant women.

Variables	PE (*n* = 12)	control (*n* = 14)	*p*
Age (years)	28.21 ± 4.51	29.36 ± 3.13	0.443
Gravida			0.596
1	5 (41.7)	6 (42.9)	
2	1 (8.3)	3 (21.4)	
≥3	6 (50.0)	5 (35.7)	
Parity			1
1	7 (58.3)	8 (57.1)	
2	5 (41.7)	6 (42.9)	
IVF	1 (8.3)	1 (7.1)	1
BMI (kg/m^2^)	26.45 ± 2.55	26.00 ± 2.71	0.67
Weight gain	16.11 ± 8.46	15.00 ± 3.85	0.663
Headache	5 (41.7)	0 (0)	0.012
Gestational age	31.5 (30, 35.75)	38.5 (38, 39)	0
SBP (mm Hg)	165.33 ± 17.87	108.14 ± 7.98	0
DBP (mm Hg)	108.42 ± 12.02	72.43 ± 6.79	0
Urinary protein (g/24h)	4.59 (2.03, 9.21)	–	
Hb (g/L)	120.83 ± 14.92	106.93 ± 14.26	0.023
PLT	183.42 ± 63.55	253.29 ± 44.28	0.003
ALB (g/L)	28.74 ± 4.25	36.36 ± 2.34	0
ALT (U/L)	10.85 (9.18, 23.08)	7.85 (5.98, 9.80)	0.009
AST (U/L)	16.90 (14.08, 28.80)	14.00 (12.00, 15.58)	0.024
Urea (mmol/L)	6.11 ± 1.72	3.40 ± 0.97	0
Cr (umol/L)	68.75 ± 17.25	48.93 ± 9.28	0.01
Neonatal weight	1705.00 ± 542.81	3295.71 ± 374.47	0
Male	7 (58.3)	7 (50)	0.976

*Data are presented as mean ± SD, median (IQR), or n (%). IVF, in vitro fertilization; BMI, body mass index; SBP, systolic blood pressure; DBP, diastolic blood pressure; Hb, hemoglobin; PLT, platelet; ALB, albumin; ALT, alanine transaminase; AST, aspartate aminotransferase; Cr, creatinine.*

### LC-MS/MS Analysis

Sample preparation included metabolite and lipid extraction was shown in Supplementary Data 1. Untargeted metabolomics and lipidomics analysis were performed using an ultra-high-performance liquid chromatography (UHPLC, 1290 Infinity LC, Agilent Technologies) in Applied Protein Technology, Shanghai, China. [See Supplementary Data 1 for further details of methodology]. To avoid systematic bias, the samples were placed in an automatic sampler with random order for continuous analysis at 4°C during the whole analysis. The pooled quality control (QC) sample was prepared by mixing equal volumes of all the samples. Insert QC samples into the sample queue to monitor and evaluate the stability of the system and the reliability of experimental data.

### Data Processing and Statistical Analysis

For clinical information, statistical analyses were conducted using SPSS v25.0 for Windows. Student’s *t*-test and the Mann–Whitney U test were used to compare continuous variables with normal and non-normal distributions between the two groups, respectively. Categorical variables were compared using the Chi-squared test or Fisher’s exact test, as appropriate. Area under receiver operating characteristic curves (AUROC) of metabolites and lipids was calculated using linear models.

The full scan and data-dependent MS2 metabolic profiles were further processed by XCMS software. Untargeted lipidomics data were processed using LipidSearch software. A supervised orthogonal partial least squares discriminant analysis (OPLS-DA) was applied to filter and classification of irrelevant noise to improve the analytical ability and validity of the model. Metabolites with variable influence on projection values (VIP) of greater than 1.0 and *p*-values of less than 0.05 were identified to be differentially expressed. The metabolic pathway analysis of these differentially expressed metabolites was performed by MetaboAnalyst website (Dhariwal et al., 2017).

A weighted metabolite co-expression network was built with R package “WGCNA” (Zhang and Horvath, 2005). Briefly, a soft thresholding power of 6 with RsquaredCut of 0.84 was chosen (Supplementary Figures 1A,B). Using these criteria, the weighted gene co-expression network analysis (WGCNA) constructed modules with minModuleSize of 30, mergeCutHeight of 0.25, deepSplit of 2 and verbose of 5. The co-expression module is a collection of metabolites with high topological overlap similarity. We calculated the correlation between the modules and the clinical traits to identify significant clinical modules. The enrichment analysis of the metabolites in the module which was related to preeclampsia also conducted in the MetaboAnalyst website (Dhariwal et al., 2017).

## Results

### Characteristics of Study Participants

Table 1 shows the characteristics of preeclampsia and normotensive pregnant women. The systolic blood pressure (SBP) of preeclampsia and normotensive pregnant women were 165.33 ± 17.87 mmHg and 108.14 ± 7.98 mmHg, and diastolic blood pressure (DBP) were 108.42 ± 12.02 mmHg and 72.43 ± 6.79 mmHg, respectively. The terminating gestational age of preeclampsia women was earlier with lower neonatal weight compared with normotensive women. The median urinary protein was 4.59 g/24 h of preeclampsia pregnant women. The age, gravida, parity, body mass index (BMI), weight gain, and sex of the fetus were comparable in the two groups. The levels of hemoglobin (Hb), alanine transaminase (ALT), aspartate aminotransferase (AST), urea, and creatinine (Cr) were higher, while albumin (ALB) and platelet (PLT) was lower in preeclampsia pregnant women compared with normotensive pregnant women.

### Metabolic Shifts in the Placenta of Preeclampsia

**FIGURE 1:**
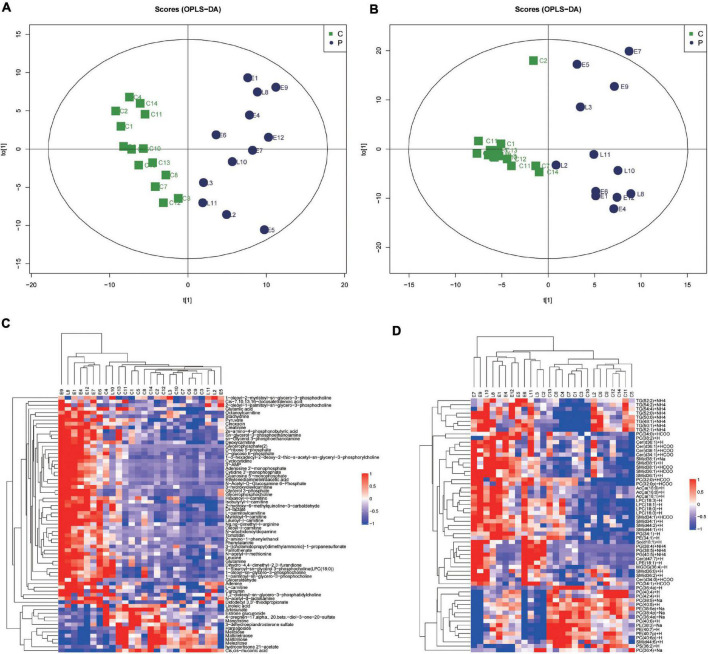
Metabolic shifts in placentas of preeclampsia. **(A,B)** The score plot of the supervised orthogonal partial least squares discriminant analysis (OPLS-DA) of metabolomic **(A)** and lipidomic analysis **(B)**. Control (green) group, preeclampsia (blue) group. Model parameters: R^2^Y = 0.845, Q^2^ = 0.351 in metabolomics analysis and R^2^Y = 0.848, Q^2^ = 0.421 in lipidomics analysis. **(C,D)** Unsupervised hierarchical clustering analysis of placental metabolome **(C)** and lipid **(D)** profiles. Each row and column represent a metabolite and a case, respectively. The relative level of each metabolite is represented by a color, as shown in the right-side color bar.

In this study, we identified 1,235 annotated metabolites and 1,545 annotated lipid species. The proportions of all kinds of metabolites were shown in Supplementary Figure 2A. The three main metabolites were lipids and lipid-like molecules (29.72%), organic acids and derivatives (20.24%), and organoheterocyclic compounds (10.12%) (Supplementary Figure 2A). There were 33 lipid classes identified in this study (Supplementary Figure 2B). According to the score plot of the supervised OPLS-DA (Figures 1A,B) of metabolites and lipid species, the preeclampsia and control groups were separated along the x-axis, early-onset preeclampsia and late-onset preeclampsia was separated along the y-axis. The key model parameters were R^2^Y = 0.845 and Q^2^ = 0.351 in metabolomics analysis and R^2^Y = 0.848 and Q^2^ = 0.421 in lipidomics analysis, indicating that the models were moderately fit. 69 metabolites (Supplementary Table 1) and 61 lipids (Supplementary Table 2) were found to be significantly different between preeclampsia and the control group (VIP > 1, *p* < 0.05). Figures 1C,D showed the hierarchical cluster heat map of samples and differential metabolites and lipid species. Most of the early-onset preeclampsia could be separated from the control group, but late-onset preeclampsia could not be separated from the control group in the metabolitic analysis. Most preeclampsia (including early-onset and late-onset preeclampsia) could be separated from the control group in lipids analysis. These results indicated that there was a significant change in metabolomics/lipidomics signatures between the preeclampsia and control groups.

### Correlation Network Analysis of Metabolites in Relation to Preeclampsia

**FIGURE 2:**
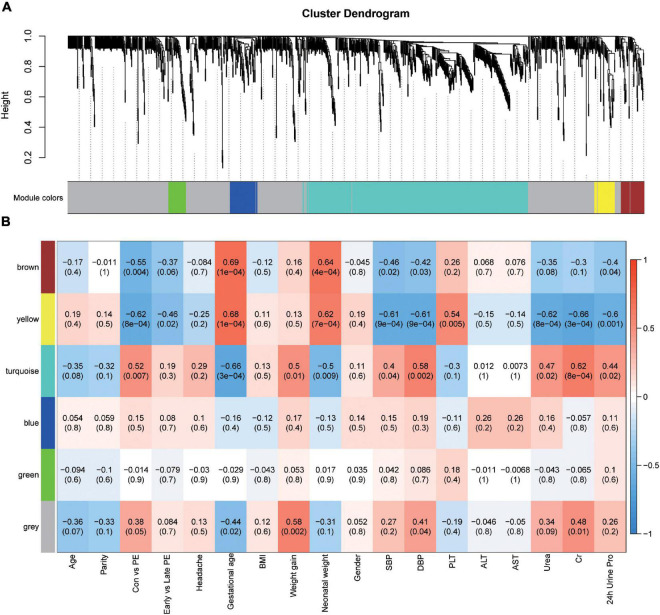
Identification of modules associated with the clinical traits of preeclampsia. **(A)** Dendrogram of all detected metabolites clustered based on the measurement of dissimilarity (1-TOM). The color band shows the results obtained from the automatic single-block analysis. **(B)** Heatmap of the correlation between the module eigengenes and clinical traits. Each row corresponds to a module eigenpeptide, column to a trait, respectively. Each cell contains the corresponding correlation and *p*-value. The table is color-coded by correlation according to the color legend.

To elucidate the relationships between metabolomics profile and clinical characteristics of pregnant women, we next constructed the correlation networks among all identified metabolites using WGCNA. Six modules were identified based on average hierarchical clustering and dynamic tree clipping (Figure 2A) in the metabolic analysis. Among the six modules, the turquoise module was highly related to preeclampsia, weight gain during pregnancy, systolic blood pressure, diastolic blood pressure, levels of urea, Cr, and the amount of urine protein in 24 h, and it was inversely related to gestational age and neonatal weight (Figure 2B). Thus, this module was selected as a clinically important module for further analysis.

### Metabolic Pathway Analysis

**FIGURE 3:**
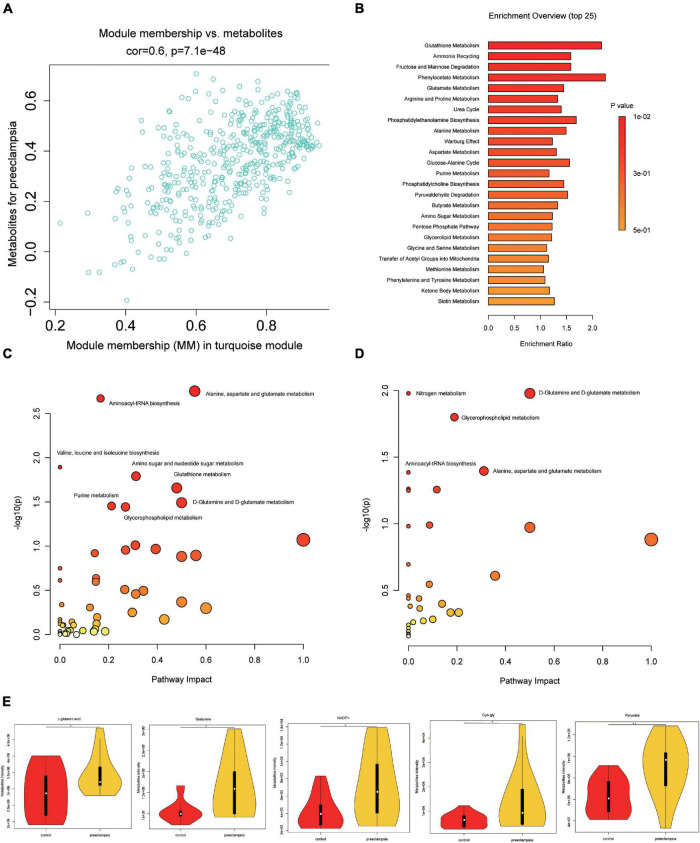
Pathway analysis of metabolites in the turquoise module and significantly altered metabolites in preeclampsia placenta. **(A)** A scatterplot of metabolites for preeclampsia vs. Module Membership (MM) in the turquoise module. **(B)** Metabolite set enrichment analysis of turquoise block using SMPDB (Small Molecule Pathway Database). The bars are colored based on their *p*-values (lower *p*-values are redder), and the bar length is based on the enrichment ratio. **(C)** Metabolomic pathway analysis of turquoise block using the KEGG database. **(D)** Metabolomic pathway analysis of significantly altered metabolites (VIP > 1, *p* < 0.05) in preeclampsia using the KEGG database. The color gradient and circle size indicate the significance of the pathway ranked by *p*-values (yellow: higher *p*-values and red: lower *p*-values) and pathway impact scores (the larger the circle, the higher the impact score), respectively. Significantly affected pathways with a low *p*-value and high pathway impact score are identified by name. **(E)** The level of L-glutamic acid, glutamine, NADP+, cys-gly and pyruvate in placentas of the control and preeclampsia groups.

The correlation between metabolites in the turquoise module and preeclampsia was shown in Figure 3A (cor = 0.6, *p* = 7.7e-48). Metabolite set enrichment analysis of turquoise module was performed using the Small Molecule Pathway Database (Figure 3B). Glutathione metabolism was enriched in the turquoise module. For a better understanding of metabolic dysregulation in preeclampsia placenta, we performed metabolic pathway analysis of the turquoise module (476 metabolites) and significantly different metabolites (69 metabolites, VIP > 1, *p* < 0.05) using the KEGG database (Figures 3C,D), separately. D-Glutamine and D-glutamate metabolism, Alanine, aspartate and glutamate metabolism, Aminoacyl-tRNA biosynthesis, and glycerophospholipid metabolism appeared to be frequently modified pathways in both analyses. L-glutamic acid and glutamine were metabolites common to all enriched pathways, which were upregulated in preeclampsia placentas (Figure 3E). Besides, pyruvate, NADP+, Cys-gly were also upregulated in preeclampsia placentas (Figure 3E). But the levels of glutathione (GSH) and glutathione disulfide (GSSG) were comparable in two groups (Supplementary Figures 3A,B).

### Lipidomic Alterations Associated With Preeclampsia

**FIGURE 4:**
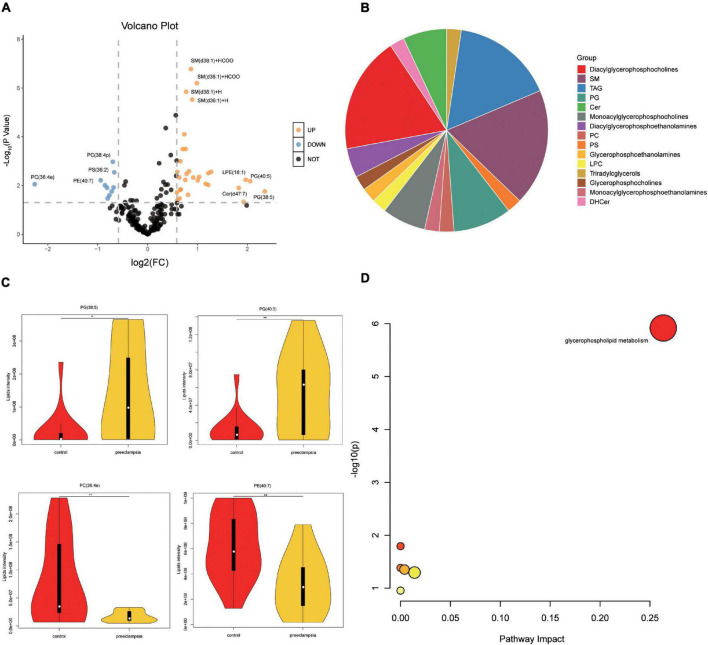
Pathway analysis of lipids in placentas. **(A)** The volcano plot of lipids (VIP > 1). In the figure, the x-axis is the log2 for fold change (FC), and the y-axis is the -Log_10_ for *p*-value. Lipids with FC > 1.5, *p*-value < 0.05 were shown in orange, and FC < 0.67, *p*-value < 0.05 are shown in blue. Others are shown in black. **(B)** The proportion of lipid subclasses of all differentially expressed lipids (VIP > 1 and *p*-value < 0.05). **(C)** The level of the top four upregulated or downregulated lipids in the control and preeclampsia group. **(D)** Pathway analysis lipids (VIP > 1, *p*-value < 0.05) using the KEGG database. The color gradient and circle size indicate the significance of the pathway ranked by *p*-values (yellow: higher *p*-values and red: lower *p*-values) and pathway impact scores (the larger the circle, the higher the impact score), respectively. Significantly affected pathways with a low *p*-value and high pathway impact score are identified by name.

We also analyzed the lipids (VIP > 1) between the preeclampsia and control group (Figure 4A and Supplementary Table 3). Lipid subclasses of the differentially expressed lipids were shown in Figure 4B. Most of the significantly changed lipids were upregulated in the preeclampsia placentas. The top four upregulated or downregulated lipids were phosphatidylglycerol (PG) (38:5), PG (40:5), phosphatidylcholines (PC) (36:4e), and phosphatidylethanolamines (PE) (40:7) with | log_2_(FC)| 2.35, 2.06, 2.25, and 0.94 (Figures 4A,C). The differential lipids were subjected to KEGG functional enrichment analysis (Figure 4D). As shown in Figure 4D, the significantly changed lipids were enriched in glycerophospholipid metabolism which was consistent with metabolic results, suggesting altered glycerophospholipid metabolism in preeclampsia placenta.

### Pathway Analysis of Combination Effect

**FIGURE 5:**
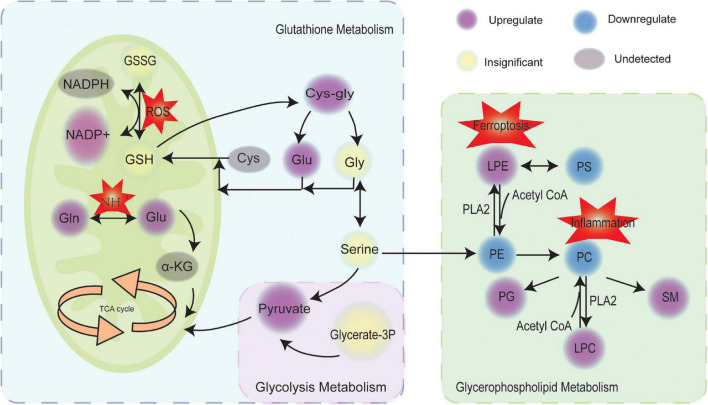
Scheme of perturbed metabolic and lipidic pathways in placenta of preeclampsia. Circles marked in purple, blue, yellow, and gray represented the upregulation, downregulation, insignificance, and undetected, respectively. GSH, glutathione; GSSG, glutathione disulfide; ROS, reactive oxygen species; Gln, glutamine; Glu, glutamate; TCA, tricarboxylic acid cycle; α-KG, α-ketoglutaric acid; Cys, cysteine; Gly, glycine; Glycerate-3P, 3-phospho-D-glycerate; PS, phosphatidylserines; PE, phosphatidylethanolamines; PC, phosphatidylcholines; PG, phosphatidylglycerol; SM, sphingomyelin; LPE, lysoPE; LPC, lysoPC; PLA2, phospholipase.

To unravel potential pathway alterations, the significantly altered metabolites and lipids detected in preeclampsia placentas were combined. A global perturbed pathway network (preeclampsia vs con) was formed with the differentially expressed metabolites and lipids (Figure 5). The glutathione metabolism pathway was closely associated with preeclampsia. Glutamate/glutamine and pyruvate, the substrates of tricarboxylic acid cycle (TCA), were increased. However, we did not discover the changes of D-glucose 1-phosphate and 3-phospho-D-glycerate (Supplementary Figure 4) between preeclampsia and normotensive placentas, both of which participated in the glycolysis pathway. In glycerophospholipid metabolism, the expression level of PC, PE, phosphatidylserines (PS) were downregulated, while lysoPE (LPE), lysoPC (LPC), and PG were upregulated in preeclampsia placentas.

### Discrimination for Preeclampsia

The receive operating characteristic (ROC) analysis of five potential metabolic and five potential lipid biomarkers (*p* < 0.05, FC > 1.5 or < 0.67, with top five of VIPs) was performed (Supplementary Table 4). The AUROC of single metabolites was near 0.8 (Supplementary Figure 5). The AUROC of combining five metabolites was 0.89 (95%CI 0.77–1.00) (Supplementary Figure 5). The AUROC of single lipid, especially SM (d36:1) + H, SM(d38:1) + H, SM(d36:1) + HCOO was near 0.99 (Supplementary Figure 6).

## Discussion

Preeclampsia is a systemic multisystem syndrome unique to pregnancy. Effective preventions and treatments are limited due to the heterogeneity and complexity of pathogenesis. A multi-omics approach combined with clinical information, which matches well with the complexity and integrity of disease, was used to provide insights into the metabolic changes of preeclampsia placenta. We found that glutathione metabolism was closely related to preeclampsia. However, there was no change in the levels of GSH and GSSG. But L-glutamate, NADP+, Cys-gly were increased in preeclampsia placentas. Besides, glycerophospholipid metabolism was significantly altered in preeclampsia placentas. These metabolic perturbations were affected with disease severity, suggesting that metabolic pathway disturbances might be associated with the pathogenesis of preeclampsia.

Weighted gene co-expression network analysis, developed by Zhang and Horvath (2005), can be used to construct a weighted gene co-expression network and form modules by similar gene expression patterns and analyze the relationship between modules and specific features (e.g., clinical information of patients) (Zhang and Horvath, 2005). The approach was also recently applied to metabolomic data analysis. In this study, we constructed a correlation network analysis of metabolites via WGCNA and identified modules with significant clinical features of PE. We found that the turquoise module was highly related to preeclampsia and the clinical traits reflecting disease severity, such as systolic blood pressure, diastolic blood pressure, levels of urea, Cr, and the amount of urine protein in 24 h, terminating gestational age and neonatal weight. Glutathione metabolism, D-Glutamine and D-glutamate metabolism, and glycerophospholipid metabolism were enriched in the turquoise module and were upregulated in preeclampsia placentas.

It was widely accepted that abnormal placentation in the first trimester was the first stage of preeclampsia which led to local hypoxia and ischemia, causing overproduction of reactive oxygen species (ROS) (Rana et al., 2019). Mitochondria finely controlled the types and levels of ROS, including antioxidants, such as GSH and glutathione peroxidases (GPXs) (Mailloux et al., 2013). GSH plays an important role in the degradation of H_2_O_2_. Two molecules of GSH are oxidized by H_2_O_2_ through glutathione peroxidase (GPX), producing glutathione disulfide (GSSG). The resulting GSSG is then reduced by glutathione reductase (GSR) and NADPH, regenerating GSH and NADP^+^ (Mailloux et al., 2013; Taysi et al., 2019). In the present study, the Metabolite set enrichment analysis of the turquoise module using the Small Molecule Pathway Database indicated that glutathione metabolism was closely related to preeclampsia. Previous studies have reported that glutathione expression in preeclampsia placenta is decreased (Ahmad et al., 2019; Zhang et al., 2020). Interestingly, in our study, both GSH and GSSG levels showed no significant differences between preeclampsia and control group, however, L-glutamate, which was a key determinant of glutathione synthesis, was significantly increased in preeclampsia placentas. Glutathione was known to be an essential endogenous antioxidant, the normal level of GSH and GSSG could be implicated in some negative feedback regulations to maintain the balance of oxidants and antioxidants. The increased levels of L-glutamate, glutamine, Cys-Gly, and NADP+ confirmed that glutathione metabolism was involved in the development of preeclampsia. The imbalance between oxidants and antioxidants in the body in which oxidation is more prone to occur may be associated with multiple organ injuries for the clinical manifestations of preeclampsia (Zhang et al., 2020).

To date, there have been only two reports of global metabolite profiling of preeclampsia placentas were performed. Austdal et al.’s (2015) study identified only 25 metabolites in the placenta by using high-resolution magic angle spinning nuclear magnetic resonance spectroscopy (HR-MAS MRS). In their study, glutamine was also significantly increased in the placenta with severe dysfunction. Consistent with our study, the ethanolamine, glycerophosphocholine, and phosphocholine lipid metabolism were abnormal in the placenta with moderate and severe dysfunction. Kaoru Kawasaki’s study by mass spectrometry-based metabolomics yielded 208 metabolites in placentas, demonstrating that early-onset preeclampsia placentas were distinct from late-onset preeclampsia and normotensive placentas (Kawasaki et al., 2019). They found that glutathione significantly changed in preeclampsia placentas versus normal placenta. However, the level of glutathione was comparable between both groups in our study. Most other changed metabolites, such as NADP+, NAD+, creatine, creatinine, glycerophosphocholine, stachydrine, ribose 5-phosphate, etc., were consistent with our results. Another study by Zhou et al. (2017), analyzed the mitochondrial metabolome in preeclampsia versus normal placenta using gas chromatography-mass spectrometry (GC-MS) analysis. In their study, glutamine, cysteine, and glycine were increased and glutathione metabolism was disturbed in mitochondria from preeclampsia placenta. Current and previous studies have suggested metabolic abnormalities in the preeclampsia placenta.

Mitochondria, particularly from trophoblastic cells, are responsible for the production of energy, which is extremely important for normal placentation. In preeclampsia placenta, mitochondrial fission/fusion appeared to be impaired (Fisher et al., 2020). More rounded, short mitochondrial profiles were observed in preeclampsia placentas with gestational age less than 34 weeks. Svoboda and Kerschbaum (2009) had reported that intramitochondrial hydrolysis of L-glutamine enhances ammonium locally and leads to mitochondrial dysfunction. It is known that glutamine is converted to glutamate in mitochondria by glutaminase (GLS) and glutamate is converted to α-ketoglutaric acid, which then participates in the TCA cycle. The TCA cycle can be fueled mainly by three substrates, fatty acids, pyruvate, and glutamine/glutamate (Solmonson and DeBerardinis, 2018). In this study, we found the level of pyruvate and glutamate were increased in preeclampsia placentas. Of note, pyruvate was the end product of the glycolysis pathway. However, we did not discover the changes of D-glucose 1-phosphate and 3-phospho-D-glycerate between preeclampsia and normotensive placentas, both of which participated in the glycolysis pathway. We assumed that reduction of oxidative phosphorylation capacity in placentas of preeclampsia partly led to a dampened mitochondrial pyruvate and glutamate uptake. Wang et al., reported that the number of mitochondria in preeclampsia placenta is far greater and these mitochondria have greater susceptibility to lipid peroxidation (Wang and Walsh, 1998). Hypoxic conditions, for example, living at an altitude, lead to susceptibility to preeclampsia (Zamudio, 2003).

In this study, the differential expressed metabolites and lipids in preeclampsia placentas were both enriched in the glycerophospholipids metabolism pathway. Glycerophospholipids are the major structural component of cell membranes (Hishikawa et al., 2014) and are involved in various biological processes including apoptosis, inflammation, and mitochondrial stress (Rodriguez-Cuenca et al., 2017). Glycerophospholipids contain a glycerol backbone which is comprised of three carbons (Ziegler and Tavosanis, 2019). Various glycerophospholipids are synthesized according to the modification of the phosphate head linking to the C3 position (Ziegler and Tavosanis, 2019). PC, the most abundant phospholipid head class, are mainly synthesized from the Kennedy pathway (Butler et al., 2020). The second most abundant phospholipid is PE, which can be generated from phosphatidylserines (PS) by headgroup exchange (Butler et al., 2020). PS is synthesized in the endoplasmic reticulum (ER) by head exchange from PC and PE (Butler et al., 2020). Choline and ethanolamine are major contributors to the production of membrane lipids (Joseph et al., 2020). Previous studies have reported that maternal choline supplementation modulates the expression of amino acid transporters and improves angiogenesis in the placenta of mice (Kwan et al., 2017). In this study, compared with the control group, the level of PC, PE and PS was downregulated, while LPE, LPC, and PG were upregulated in preeclampsia placentas. PC might convert to LPC, PG and sphingomyelin (SM) and PE might convert to LPE in preeclampsia placenta. Phospholipase PLA2 activity is increased in preeclamptic placental tissue (Austdal et al., 2015), which decomposes PC to release glycerophosphocholine and arachidonic acid, possibly playing a role in increased inflammation, a central process in the preeclamptic placenta (Austdal et al., 2015). In Austdal et al.’s (2015) metabolic profiles of placenta using high resolution magic angle spinning magnetic resonance spectroscopy (HR-MAS MRS), glycerophosphocholine was also increased in preeclampsia placentas. Zhang et al. (2020) reported that ferroptosis was involved in the pathogenesis of preeclampsia. Excessive accumulation of hydroperoxyl phosphatidylethanolamine (Hp-PE) is a characteristic of ferroptosis, which can be metabolize to LPE (Beharier et al., 2020). The mechanism of disturbed lipids on preeclampsia is not well known. The lipids metabolism of preeclampsia placentas needs to be studied further.

Based on the screening criteria for biomarkers reported by Hosseininejad et al. (2009), the discriminating power of SM (d36:1) + H, SM(d38:1) + H, or SM(d36:1) + HCOO is excellent, there is no need to combine other lipids or metabolites. It indicates that adding some lipid-related indicators may be helpful to improve the predictive of preeclampsia. SM is one of the important classes of phospholipids essential for cell membrane function (Al-Sari et al., 2021). SM content of the membrane directly affects cholesterol homeostasis. Researches showed that degradation of SM in cultured cells leads to cholesterol translocation from the plasma membrane to the ER (Slotte, 2013). Plasma SM levels was an independent risk factor for coronary heart disease in humans (Schlitt et al., 2006). SM levels have also been associated with mild-to-moderate hypertension (Shahin et al., 2017). SM may participate the pathophysiological process of preeclampsia, though the mechanism is not known. These results also confirmed that there were significant changes in metabolites and lipids in the preeclampsia placenta, which might provide some new insight into the pathogenesis of preeclampsia. As the discriminating power of models might be overestimated, external validation was needed. Maternal plasma from early gestational age is needed to examine whether these metabolites and lipids have the ability to predict the occurrence of preeclampsia further.

### Limitation

A limitation of our study is that the sample size was small, which lead to some of the differential metabolites being undetected. Therefore, large-scale validation or prospective studies are needed. Although some significant metabolites were identified, there were still many metabolites unidentified due to the limited number of reliable spectral reference databases for metabolite identification. This is a cross-sectional study that cannot draw a causal inference about the association between differential metabolites and preeclampsia. The differential metabolites found in this study and potential mechanism are needed to be further validated *in vivo* or *in vitro*.

## Conclusion

In summary, we used UHPLC to characterize the placental metabolomics and lipidomics profiling for women complicated with preeclampsia. There were metabolic and lipidomic disturbances in both early-onset and late-onset preeclampsia placenta. The metabolic perturbations were primarily associated with glycerophospholipid and glutathione metabolism. The former might influent membrane structures of organisms, and then cell behaviors. The latter might inversely influent glutamate metabolism, and then mitochondria function. Our research highlighted some important mechanisms involved in the pathophysiological changes of preeclampsia.

## Data Availability Statement

The original contributions presented in the study are included in the article/Supplementary Material, further inquiries can be directed to the corresponding authors.

## Ethics Statement

The studies involving human participants were reviewed and approved by The Third Affiliated Hospital of Guangzhou Medical University. The patients/participants provided their written informed consent to participate in this study.

## Author Contributions

LZ and SB: conceptualization, samples collection, analysis, and writing. YLi, LH, YLia, MH, BH, JL, WD, and SG: samples collection, editing, and analysis. JC, LD, DC, and ZW: supervision, project administration, and funding acquisition. All authors read and approved the final manuscript.

## Conflict of Interest

The authors declare that the research was conducted in the absence of any commercial or financial relationships that could be construed as a potential conflict of interest.

## Publisher’s Note

All claims expressed in this article are solely those of the authors and do not necessarily represent those of their affiliated organizations, or those of the publisher, the editors and the reviewers. Any product that may be evaluated in this article, or claim that may be made by its manufacturer, is not guaranteed or endorsed by the publisher.

## Supplementary Material

Supplementary Figure 1 Determination of soft-threshold power in the WGCNA. **(A)** Analysis of the scale-free index for various soft-threshold powers (β). **(B)** Analysis of the mean connectivity for various soft-threshold powers.

Supplementary Figure 2 **(A)** The proportion of metabolites subclasses. **(B)** Thirty-three lipid classes identified in this study.

Supplementary Figure 3 **(A,B)** The expression of GSH and GSSG in control and preeclampsia group.

Supplementary Figure 4 **(A,B)** The expression of D-glucose 1-phosphate and 3-phospho-D-glycerate in control and preeclampsia group.

Supplementary Figure 5 **(A–D)** The ROC curve of Glycerophosphocholine, L-palmitoylcarnitine, Maltotriose, and five metabolites.

Supplementary Figure 6 **(A–C)** The ROC curve of SM(d36:1) + H, SM (d38:1) + H, and SM(d36:1) + HCOO.
