# Supplementary material for: Integrated Metabolomic and Lipidomic Analysis in the Placenta of Preeclampsia
Source: Front Physiol. 2022 Feb 4;13:807583. doi: 10.3389/fphys.2022.807583 (PMC8854797; doi:10.3389/fphys.2022.807583)

Supplementary Material

1 Supplementary Data

Sample preparation

Metabolite Extraction

1 mL of pre-cold extraction solvent methanol/acetonitrile/water (2:2:1, v/v/v) was added to 80 mg sample, followed by vortex. The lysate was sonicated at 4°C (30 min/once, twice) followed by sitting still for 10 min at -20°C， then centrifuged at 14,000 g for 20 min at 4°C and the supernatant was dried in a vacuum centrifuge. For LC-MS analysis, the samples were re-dissolved in 100 μL acetonitrile/water (1:1, v/v) solvent.

Lipid Extraction

30 mg sample with 200 µL of water and appropriate magnetic beads were flash-frozen in liquid nitrogen for 5 s, and then was homogenized using MP homogenizer (24×2, 6.0 M/S, 60 s) for three times. 240 µL of pre-cold methyl alcohol was added, followed by vortex, and 800 µL of MTBE was added and the mixture was sonicated at 4°C for 20 min followed by sitting still for 30 min at room temperature. The solution was centrifuged at 14,000 g for 15 min at 10°C and the upper organic solvent layer was obtained and dried under nitrogen.

LC-MS/MS Analysis

Untargeted Metabolomics Analysis

Analysis was performed using an ultra-high-performance liquid chromatography (UHPLC, 1290 Infinity LC, Agilent Technologies) coupled to a quadrupole time-of-flight (AB Sciex TripleTOF 6600) in Shanghai Applied Protein Technology Co.,Ltd.

For hydrophilic interaction liquid chromatography (HILIC) separation, samples were analyzed using a 2.1 mm × 100 mm ACQUIY UPLC BEH 1.7 µm column (waters, Ireland) with column temperature 25 ℃, flow rate 0.5 mL/min, and injection volume 2 μL. The mobile phase contained, A: 25 mM ammonium acetate, 25 mM ammonium hydroxide and water, B: acetonitrile. The samples were eluted using linear-gradient. The gradient was 95% B for 0.5 min and was linearly reduced to 65% in 7 min, and then was reduced to 40% in 8 min and kept for 1 min. Then it was increased to 95% in 9.1min and kept for 3min.

AB Triple TOF 6600 mass spectrometer (AB SCIEX) adopt electrospray ionization (ESI) positive and negative ion modes for detection to collect the first and second order spectrograms of samples. The parameters of ESI source were set as follows: Ion Source Gas1 (Gas1) of 60, Ion Source Gas2 (Gas2) of 60, curtain gas (CUR) of 30 psi, ion source temperature of 600℃, Ion Spray Voltage Floating (ISVF) of ± 5500 V. In MS only acquisition, the instrument was set to acquire over the m/z range 60-1000 Da, and the accumulation time for TOF MS scan was set at 0.20 s/spectra. In auto MS/MS acquisition, the instrument was set to acquire over the m/z range 25-1000 Da, and the

accumulation time for product ion scan was set at 0.05 s/spectra. Information dependent acquisition (IDA) with high sensitivity mode selected was used to acquire the product ion scan. The parameters were set as follows: the collision energy (CE) fixed at 35 ± 15 eV; declustering potential (DP) of 60 V (+) and −60 V (−); exclude isotopes within 4 Da, candidate ions to monitor per cycle of 10.

Untargeted Lipidomics Analysis.

Analysis was performed using an ultra-high-performance liquid chromatography (UHPLC, Nexera LC-30A) with column temperature 45 °C, flow rate 300 μL/min. The mobile phase contained, A: acetonitrile-water (6:4, v/v) with 10 mM ammonium formate; B: acetonitrile–isopropanol (1:9, v/v) with 10 mM ammonium formate. The separation gradient was optimized as follows: initial 30% solvent B held for 2 min ramping to 100% for 23 min, followed by equilibrating at 30% solvent B for 10 min.

Mass spectra was acquired by Q-Exactive Plus ESI+ and ESI- detection modes with identical ionization parameters including heater temperature of 300°C, sheath gas flow rate of 45 arb, auxiliary gas flow rate of 15 arb, sweep gas flow rate of 1 arb, capillary temperature of 350°C, except spray voltage, S-Lens RF level and MS1 scan ranges (3.0 KV, 50%, 200-1800 for the ESI+ mode and 2.5 KV, 60%, 250-1800 for the ESI-mode, respectively). Lipids were structurally identified through acquiring data-dependent MS2 spectra, and the resolution is 70,000 at m/z = 200 (MS1) and 17,500 at m/z =200 (MS2).

2 Supplementary Tables

Table S1 69 significantly different metabolites between preeclampsia and the control group (VIP>1, *P*<0.05)

Name	VIP	P	FC
Melibiose	2.025446	0.000222	0.457607
Maltotriose	4.914621	0.002717	0.526362
Melezitose	1.340238	0.003295	0.617647
Glycerophosphocholine	12.88151	0.001736	3.686033
L-palmitoylcarnitine	5.629242	0.001492	3.078361
Stachydrine	1.120234	0.001422	5.285549
2-oleoyl-1-palmitoyl-sn-glycero-3-phosphocholine	4.803059	0.003981	1.197565
hydrocortisone 21-acetate	1.298565	0.018174	0.497236
Lauroyl-l-carnitine	1.033033	0.003466	2.210009
Myristoyl-l-carnitine	2.000766	0.007541	2.497738
1-Stearoyl-sn-glycerol 3-phosphocholine(LPC(18:0))	1.803334	0.026592	1.415899
Tomatidin	1.911097	0.012078	3.994603
1-palmitoyl-sn-glycero-3-phosphocholine	4.208268	0.018033	1.554407
Cyclocytidine	1.017705	0.004535	5.264934
1-o-hexadecyl-2-deoxy-2-thio-s-acetyl-sn-glyceryl-3-phosphorylcholine	7.249508	0.004688	3.255282
3-hydroxyoleylcarnitine	3.419497	0.012329	3.87471
Oleoyl-l-carnitine	3.71862	0.021881	3.540988
L-carnitine	1.105559	0.016599	1.307242

1,2-dioleoyl-sn-glycero-3-phosphatidylcholine	2.416655	0.029747	1.143134
N-arachidonoyldopamine	1.221366	0.021041	3.591612
Sn-glycerol-3-phosphoethanolamine	1.117901	0.020102	2.220544
Adenosine 3'-monophosphate	1.908392	0.013506	3.53146
3-[(cholamidopropyl)dimethylammonio]-1-propanesulfonate	1.104246	0.024782	2.032164
Octanoylcarnitine	1.046861	0.015306	1.894968
1-oleoyl-sn-glycero-3-phosphocholine	1.218372	0.048206	1.567782
Hexanoyl-l-carnitine	2.14557	0.013463	1.616877
Cinoxacin	1.04375	0.006727	1.688025
Creatinine	1.137406	0.001229	1.499928
1-oleoyl-2-myristoyl-sn-glycero-3-phosphocholine	1.356899	0.007365	1.293397
2-amino-1-phenylethanol	1.246317	0.04152	1.479684
Deoxycarnitine	1.455667	0.017795	1.493513
Didodecyl 3,3'-thiodipropionate	1.191478	0.035195	1.168035
Ethylenediaminetetraacetic acid	2.089929	0.032894	4.279471
Isobutyryl-l-carnitine	2.424867	0.025878	1.615585
N-acetyl-d-lactosamine	1.106843	0.007758	1.481222
Ng,ng-dimethyl-l-arginine	3.041429	0.020224	1.627952
Artesunate	2.574547	5E-05	0.38451
Harpagoside	1.282402	0.002183	0.562741
Mangostine	1.501904	0.004064	0.512366
Adenine	1.195001	0.000601	2.481173
Maltotetraose	1.518507	0.014347	0.607933
Glycerol 3-phosphate	1.45786	0.002284	3.454552
Cis,cis-muconic acid	7.542896	0.014628	0.859347
3-dehydroepiandrosterone sulfate	2.797491	0.025432	0.668733
Cis-7,10,13,16-docosatetraenoic acid	1.253796	0.012776	1.279698
Estrone glucuronide	1.497301	0.022816	0.35713
4-pregnen-17.alpha., 20.beta.-diol-3-one-20-sulfate	1.587464	0.028789	0.575582
2-hydroxy-6-methylquinoline-3-carbaldehyde	2.717551	0.00175	1.80784
Glyceraldehyde	1.780404	0.004893	2.923781
Dl-lactate	4.485758	0.002116	1.796609
Guanosine 5'-monophosphate	2.121229	0.010069	3.431666
Cytidine 3'-monophosphate	2.831636	0.008275	3.198497
Glycerophosphate(2)	1.857486	0.025204	7.330373
3'-AMP	2.500772	0.019137	3.486563
sn-Glycerol 3-phosphoethanolamine	5.073493	0.015045	2.106261
N-acetyl-l-methionine	1.066881	0.013966	1.910433
N-Acetyl-D-Glucosamine 6-Phosphate	1.996654	0.03521	2.670377
D-glucose 6-phosphate	3.477637	0.027075	2.463948
D-ribose 5-phosphate	1.22561	0.024552	2.191176
2s-amino-4-phosphonobutyric acid	1.174775	0.018618	1.955985
Pantothenate	2.233923	0.025364	1.864588
Curcumin	3.583864	0.028451	1.383734

Dihydro-4,4-dimethyl-2,3-furandione	1.100852	0.009798	1.525402
Glutamic acid	1.837953	0.04472	1.199945
Glutamine	2.227944	0.010296	1.531501
Leucine	3.432427	0.022743	1.401908
Linoleic acid	4.143512	0.041779	0.876417
Phenylalanine	1.776071	0.037741	1.613426
Pyruvate	1.683424	0.004321	1.436721

Table S2 61significantly different lipids between preeclampsia and the control group (VIP>1, $P<0.05$)

LipidGroup	VIP	P	FC
Cer(d38:1)+HCOO	1.596151	0.000957	1.497184
PE(34:1)-H	1.127962	0.025868	1.197246
PG(34:1)-H	4.801422	0.004028	1.486645
SM(d34:1)+HCOO	4.146145	4.39E-05	1.286863
PC(32:0p)+HCOO	1.279767	0.019586	1.294536
SM(d36:1)+HCOO	2.680738	6.26E-07	1.981293
MGDG(36:4)-H	1.17721	0.002619	1.776852
PC(32:0)+HCOO	3.789803	0.014335	1.210803
SM(d38:1)+HCOO	2.359008	1.56E-07	1.82663
PC(34:1)+HCOO	2.062617	0.033363	1.136978
PC(34:0)+HCOO	1.135685	0.007184	1.246428
AcCa(16:0)+H	2.524171	0.003114	2.326661
SM(d34:1)+H	8.301511	0.001223	1.173415
AcCa(18:1)+H	1.47198	0.008496	2.239018
SM(d36:2)+H	1.653532	0.00913	1.319876
SM(d36:1)+H	5.638267	2.98E-06	1.857049
SM(d38:1)+H	5.679035	1.42E-06	1.707979
PE(40:7p)+H	1.921383	0.017486	0.608019
SM(d38:1)+Na	1.645054	1.32E-05	1.478859
LPC(16:0)+H	4.295753	0.004447	2.045191
PE(40:7)+H	3.033355	0.006098	0.520825
PE(38:3)+Na	2.67904	0.012415	0.569538
PG(38:5)+NH4	1.954762	0.017556	5.091006
PG(38:4)+NH4	2.166015	0.047476	3.795593
PC(40:6p)+H	1.595423	0.009879	0.553475
LPC(18:3)+H	1.675546	0.002756	2.41595
LPC(18:1)+H	1.210715	0.005934	1.688152
PC(38:5)+Na	1.960477	0.006925	0.737694
LPC(18:0)+H	2.06143	0.004726	1.8854
PC(40:6)+H	1.95464	0.025484	0.587935
PC(40:5)+H	1.466038	0.048689	0.731947
PC(40:4)+H	1.7962	0.012019	0.623246

TG(50:0)+NH4	2.364599	0.00333	1.717427
LPE(18:1)-H	1.015462	0.005811	3.912439
Cer(d34:0)+HCOO	1.081067	0.034012	1.559784
Cer(d36:1)+HCOO	2.422138	7.9E-05	1.665126
Cer(d47:7)+H	1.444015	0.012553	3.543084
AcCa(18:0)+H	1.301782	0.009514	2.322102
SM(d36:0)+H	1.179395	0.00281	1.511897
PC(36:4e)+H	1.241709	0.00888	0.208402
PE(38:6e)+Na	1.062625	0.009845	0.724726
PS(36:2)+H	1.415963	0.00287	0.629158
PC(36:4e)+Na	1.533488	0.041471	0.81487
PC(36:4)+Na	1.719425	0.040572	0.788663
PC(38:2)+H	1.592316	0.004677	1.554193
PC(38:4p)+Na	1.656019	0.001069	0.616603
TG(48:1)+NH4	1.464284	0.04077	1.510363
So(d18:1)+H	1.171395	0.006145	2.006086
SM(d44:6)+H	1.368422	0.042622	0.858368
SM(d44:2)+H	1.157049	0.000567	1.379357
PG(40:5)+NH4	1.163143	0.006638	4.156031
SM(d44:1)+H	1.433706	0.001006	1.576623
TG(50:1)+NH4	3.682934	0.01481	1.580093
PC(42:4)+H	1.092033	0.03404	0.574378
TG(52:2)+NH4	3.115639	0.015128	1.386235
TG(52:1)+NH4	2.058054	0.014961	1.573958
TG(52:0)+NH4	1.129284	0.000319	1.626118
TG(54:4)+NH4	1.466573	0.024075	1.752484
TG(54:2)+NH4	1.460397	0.019472	1.502716
Cer(d36:1)+H	1.46286	0.000316	1.694014
Cer(d34:1)+HCOO	3.359891	0.009273	1.281798

Table S3 Lipids (VIP＞1) between the preeclampsia and control group.

LipidGroup	VIP	P	FC
Cer(d38:1)+HCOO	1.596151	0.000957	1.497184
PE(34:1)-H	1.127962	0.025868	1.197246
PG(34:1)-H	4.801422	0.004028	1.486645
SM(d34:1)+HCOO	4.146145	4.39E-05	1.286863
PC(32:0p)+HCOO	1.279767	0.019586	1.294536
PE(40:7p)-H	1.491134	0.067954	0.68196
SM(d36:1)+HCOO	2.680738	6.26E-07	1.981293
MGDG(36:4)-H	1.17721	0.002619	1.776852
PC(32:0)+HCOO	3.789803	0.014335	1.210803
PE(40:7)-H	2.758018	0.067156	0.611891
PE(40:6)-H	2.240954	0.070513	0.691221
SM(d38:1)+HCOO	2.359008	1.56E-07	1.82663

PC(34:1)+HCOO	2.062617	0.033363	1.136978
PC(34:0)+HCOO	1.135685	0.007184	1.246428
PC(38:4p)+HCOO	1.23286	0.10011	0.708705
AcCa(16:0)+H	2.524171	0.003114	2.326661
SM(d34:1)+H	8.301511	0.001223	1.173415
AcCa(18:1)+H	1.47198	0.008496	2.239018
PE(34:1)+H	1.260502	0.069596	1.141791
SM(d36:2)+H	1.653532	0.00913	1.319876
SM(d36:1)+H	5.638267	2.98E-06	1.857049
PE(36:4p)+Na	1.526609	0.072124	0.808446
PC(34:4)+H	1.332469	0.092365	1.399925
SM(d38:1)+H	5.679035	1.42E-06	1.707979
PC(36:4p)+H	4.961513	0.080231	0.76723
PE(40:7p)+H	1.921383	0.017486	0.608019
SM(d38:1)+Na	1.645054	1.32E-05	1.478859
LPC(16:0)+H	4.295753	0.004447	2.045191
PE(40:8)+H	1.344727	0.084888	0.5932
PE(40:7)+H	3.033355	0.006098	0.520825
PE(38:3)+Na	2.67904	0.012415	0.569538
PC(38:6e)+H	1.585626	0.082249	0.699298
PC(38:4p)+H	4.558928	0.078567	0.699142
PC(38:5)+H	7.656397	0.078948	0.768253
PG(38:5)+NH4	1.954762	0.017556	5.091006
PG(38:4)+NH4	2.166015	0.047476	3.795593
PG(38:3)+NH4	2.042703	0.06558	3.958206
PC(40:6p)+H	1.595423	0.009879	0.553475
LPC(18:3)+H	1.675546	0.002756	2.41595
LPC(18:1)+H	1.210715	0.005934	1.688152
PC(38:6)+Na	2.320738	0.087351	0.719048
PC(38:5)+Na	1.960477	0.006925	0.737694
LPC(18:0)+H	2.06143	0.004726	1.8854
PC(38:4)+Na	1.480365	0.10487	0.83039
PC(40:6)+H	1.95464	0.025484	0.587935
PC(40:5)+H	1.466038	0.048689	0.731947
PC(40:4)+H	1.7962	0.012019	0.623246
TG(50:0)+NH4	2.364599	0.00333	1.717427
LPE(18:1)-H	1.015462	0.005811	3.912439
LPC(16:0)+HCOO	1.271802	0.070778	1.599195
Cer(d34:0)+HCOO	1.081067	0.034012	1.559784
Cer(d36:1)+HCOO	2.422138	7.9E-05	1.665126
Cer(d40:1)+HCOO	1.394945	0.473485	1.093042
Cer(d42:2)+HCOO	1.067975	0.367893	1.110218
Cer(d42:1)+HCOO	1.686645	0.556465	1.088269
PE(36:4p)-H	3.184994	0.671723	0.932986

PE(36:4)-H	2.054992	0.702841	0.93173
PE(38:6p)-H	3.101758	0.226072	0.801895
PE(38:5p)-H	2.178466	0.797194	0.946186
PE(38:4p)-H	3.456157	0.662435	0.924862
PE(38:4p)-H	1.780242	0.403042	0.862955
PE(38:6)-H	1.067746	0.876248	0.967565
PE(38:5)-H	1.605146	0.780442	0.943712
PC(32:0e)+HCOO	1.049276	0.107535	1.186056
PE(38:4)-H	3.259155	0.494407	0.890262
PE(38:3)-H	1.169393	0.691341	1.084698
DG(36:4)+NH4	1.350832	0.793288	0.94377
PE(40:6p)-H	2.104836	0.332565	0.83312
PS(35:0)-H	1.136164	0.092613	1.454499
PE(40:4p)-H	1.078396	0.485477	0.860487
PC(34:2)+HCOO	2.280145	0.063481	1.22758
Cer(d42:5)+H	1.116577	0.05797	0.741202
PC(36:4p)+HCOO	1.275584	0.217785	0.810712
Cer(d42:4)+H	1.174616	0.147605	0.873515
PS(39:3)-H	2.175526	0.741304	1.044803
PC(36:3)+HCOO	1.188098	0.511382	1.111957
PC(36:2)+HCOO	1.561726	0.071948	1.275938
SM(d40:1)+HCOO	2.099222	0.060369	1.193819
PS(40:6)-H	1.071442	0.113619	0.838459
PC(38:6)+HCOO	1.985361	0.140476	0.701489
PC(38:5)+HCOO	1.907221	0.325873	0.844088
PS(41:3)-H	1.631204	0.69542	1.062902
PC(38:3)+HCOO	1.353774	0.060935	1.396838
PI(36:4)-H	1.406297	0.83882	0.94141
SM(d42:2)+HCOO	1.03734	0.072803	1.14453
SM(d42:1)+HCOO	1.253605	0.67207	1.046503
Cer(d42:1)+H	1.035157	0.950747	0.988226
PI(38:4)-H	2.620403	0.41898	0.820757
DG(38:4)+NH4	1.892583	0.166601	0.771101
Cer(d42:1)+Na	1.589501	0.103419	0.844795
SM(d32:1)+H	1.333447	0.08451	1.206607
SM(d34:2)+H	1.168882	0.9278	0.989811
SM(d34:0)+H	1.841704	0.222388	1.162222
PC(30:0)+H	2.115093	0.270291	1.136153
Cer(d47:7)+H	1.444015	0.012553	3.543084
PE(34:2)+H	1.014771	0.233476	1.198806
AcCa(18:0)+H	1.301782	0.009514	2.322102
PC(32:1e)+H	3.60243	0.209405	1.142834
PC(31:0)+H	1.354488	0.605171	1.06848
PC(32:0e)+H	3.057721	0.367278	1.100738

PE(34:2e)+Na	4.36811	0.512033	0.894963
SM(d34:1)+Na	1.332394	0.45779	0.96439
PC(32:1)+H	2.751545	0.156276	1.194685
SM(d36:0)+H	1.179395	0.00281	1.511897
PC(32:0)+H	8.196151	0.088832	1.101717
PE(34:1)+Na	2.643284	0.581182	0.901202
PC(32:1e)+Na	1.228062	0.529672	1.108057
PE(36:3)+H	1.105027	0.582542	1.127305
PC(33:2)+H	1.023094	0.325678	1.143099
PC(34:1e)+H	2.039002	0.916489	1.012048
PE(36:4e)+Na	3.392043	0.171553	0.775796
PE(38:5p)+H	3.059966	0.277564	0.820584
PE(38:5p)+H	1.253643	0.524514	0.863975
PE(36:2e)+Na	4.070215	0.67688	0.927579
PE(38:4p)+H	2.21357	0.290715	0.830617
PC(34:4)+H	1.046942	0.391312	1.20354
PC(34:3)+H	1.136704	0.454419	0.883985
PC(34:2)+H	5.235214	0.141887	1.072991
PC(34:1)+H	5.747621	0.274634	1.07004
PC(34:0)+H	3.29417	0.075149	1.161981
PE(38:6)+H	1.275644	0.375883	0.837936
PE(36:3)+Na	1.076315	0.634763	0.877973
PC(35:5)+H	2.2276	0.533717	0.884364
PC(36:4p)+H	1.47988	0.286782	0.813876
PC(35:4)+H	4.569105	0.449658	0.879169
PC(36:4e)+H	3.694378	0.523207	0.898101
PC(36:4e)+H	1.241709	0.00888	0.208402
PE(36:0)+Na	1.747667	0.982125	1.004893
PE(38:6e)+Na	1.062625	0.009845	0.724726
PE(38:4p)+Na	1.55793	0.170337	0.821549
PE(38:4e)+Na	2.699344	0.173536	0.765212
PC(34:2)+Na	2.229973	0.502525	1.049597
PC(36:5)+H	1.534819	0.432712	1.18895
PC(36:5)+H	1.058265	0.665174	1.103595
PE(40:4p)+H	1.015488	0.80267	0.946111
PC(36:4)+H	1.041263	0.523761	0.936883
PC(36:4)+H	6.211649	0.968019	0.995805
PC(34:1)+Na	1.33284	0.931512	1.019268
PC(36:4)+H	3.004881	0.780666	0.934125
PC(36:3)+H	5.44508	0.157745	1.213926
PC(36:3)+H	2.375892	0.154221	1.212081
SM(d40:2)+H	1.102909	0.954509	0.993278
SM(d40:1)+H	5.146012	0.13136	1.153048
PS(36:2)+H	1.415963	0.00287	0.629158

PC(36:1)+H	2.761099	0.910353	0.989126
PE(38:4)+Na	1.123708	0.290465	0.848049
PC(36:4e)+Na	1.533488	0.041471	0.81487
PC(38:6p)+H	1.232908	0.300162	0.785325
PE(38:2)+Na	1.120937	0.279674	0.787302
PC(38:4p)+H	1.233786	0.086117	0.670722
PC(38:4p)+H	1.695525	0.321018	0.832381
PC(37:4)+H	1.679031	0.202198	0.814491
PE(38:1)+Na	1.318325	0.1794	0.777413
PC(38:4e)+H	1.100317	0.139164	0.78105
PC(38:4e)+H	2.109792	0.292753	0.828555
SM(d41:1)+H	1.664289	0.47482	1.103065
PC(36:4)+Na	1.719425	0.040572	0.788663
PC(38:6)+H	3.747023	0.631646	0.903978
PC(38:6)+H	7.111	0.132819	0.700478
So(d18:2)+H	1.006484	0.150901	1.548621
SM(d42:5)+H	1.005	0.240101	0.874347
PC(38:5)+H	2.385613	0.192727	0.829051
SM(d40:1)+Na	1.2556	0.225894	1.043693
PC(38:4)+H	4.982522	0.888094	0.980421
PC(36:1)+Na	1.495725	0.132532	0.886915
PC(38:4)+H	2.178105	0.998559	1.000314
SM(d42:3)+H	1.728482	0.624073	0.954489
PS(38:4)+H	1.284912	0.120535	0.648224
PC(36:0)+Na	3.526995	0.151179	1.269716
PC(38:3)+H	1.465814	0.235601	1.231631
SM(d42:2)+H	2.664902	0.205133	1.095105
PC(38:2)+H	1.592316	0.004677	1.554193
SM(d42:1)+H	4.224786	0.716418	1.045919
PC(38:4p)+Na	1.656019	0.001069	0.616603
Cer(d32:0)+H	1.150784	0.122586	0.790627
TG(48:1)+NH4	1.464284	0.04077	1.510363
TG(48:0)+NH4	2.248081	0.052063	1.503115
PC(40:9)+H	1.491278	0.306834	0.858006
PC(40:8)+H	2.767355	0.66971	0.889721
PC(40:7)+H	2.29227	0.566772	0.856327
PC(40:7)+H	2.202368	0.235114	0.762895
So(d18:1)+H	1.171395	0.006145	2.006086
SM(d44:6)+H	1.368422	0.042622	0.858368
PC(40:6)+H	2.137258	0.631215	0.907709
SM(d42:2)+Na	1.170675	0.78046	0.984157
PC(40:5)+H	1.848103	0.199195	0.791216
SM(d44:2)+H	1.157049	0.000567	1.379357
PG(40:5)+NH4	1.163143	0.006638	4.156031

SM(d44:1)+H	1.433706	0.001006	1.576623
TG(50:2)+NH4	1.822644	0.145626	1.38748
TG(50:1)+NH4	3.682934	0.01481	1.580093
PC(42:10)+H	2.27557	0.240327	0.643725
PC(42:9)+H	1.098982	0.267234	0.670415
Cer(d34:1)+H	2.029738	0.102045	1.214771
PC(42:4)+H	1.092033	0.03404	0.574378
TG(52:4)+NH4	1.019422	0.094519	1.498627
TG(52:4)+NH4	1.114853	0.617668	1.146509
TG(52:3)+NH4	2.181138	0.320383	1.258862
TG(52:2)+NH4	3.115639	0.015128	1.386235
TG(52:1)+NH4	2.058054	0.014961	1.573958
TG(52:0)+NH4	1.129284	0.000319	1.626118
TG(54:5)+NH4	1.167975	0.149885	1.517964
TG(54:4)+NH4	1.107927	0.618003	1.15619
TG(54:4)+NH4	1.466573	0.024075	1.752484
TG(54:3)+NH4	1.571057	0.374031	1.149593
PI(38:4)+NH4	1.131927	0.408998	0.808301
TG(54:2)+NH4	1.460397	0.019472	1.502716
TG(56:5)+NH4	1.083767	0.453766	1.177688
Cer(d36:1)+H	1.46286	0.000316	1.694014
Cer(d32:1)+HCOO	1.119001	0.056491	1.202708
Cer(d34:2)+HCOO	1.456984	0.294673	1.100681
Cer(d34:1)+HCOO	3.359891	0.009273	1.281798

Table S4 The ROC analysis of 5 potential metabolic and 5 potential lipid biomarkers.

Name	VIP	P	FC	AUC	95% CI
**Metabolites**					
Glycerophosphocholine	12.88151	0.001736	3.686033	0.8	0.62-0.98
L-palmitoylcarnitine	5.629242	0.001492	3.078361	0.82	0.63-1
sn-Glycerol 3-phosphoethanolamine	5.073493	0.015045	2.106261	0.77	0.58-0.96
Maltotriose	4.914621	0.002717	0.526362	0.83	0.68-0.99
Dl-lactate	4.485758	0.002116	1.796609	0.73	0.50-0.96
**Lipids**					
SM(d38:1)+H	5.679035	1.42E-06	1.707979	0.99	0.96-1
SM(d36:1)+H	5.638267	2.98E-06	1.857049	0.99	0.96-1
LPC(16:0)+H	4.295753	0.004447	2.045191	0.83	0.68-0.99
TG(50:1)+NH4	3.682934	0.01481	1.580093	0.78	0.60-0.96
SM(d36:1)+HCOO	2.680738	6.26E-07	1.981293	0.99	0.98-1

3 Supplementary Figures

Figure S1

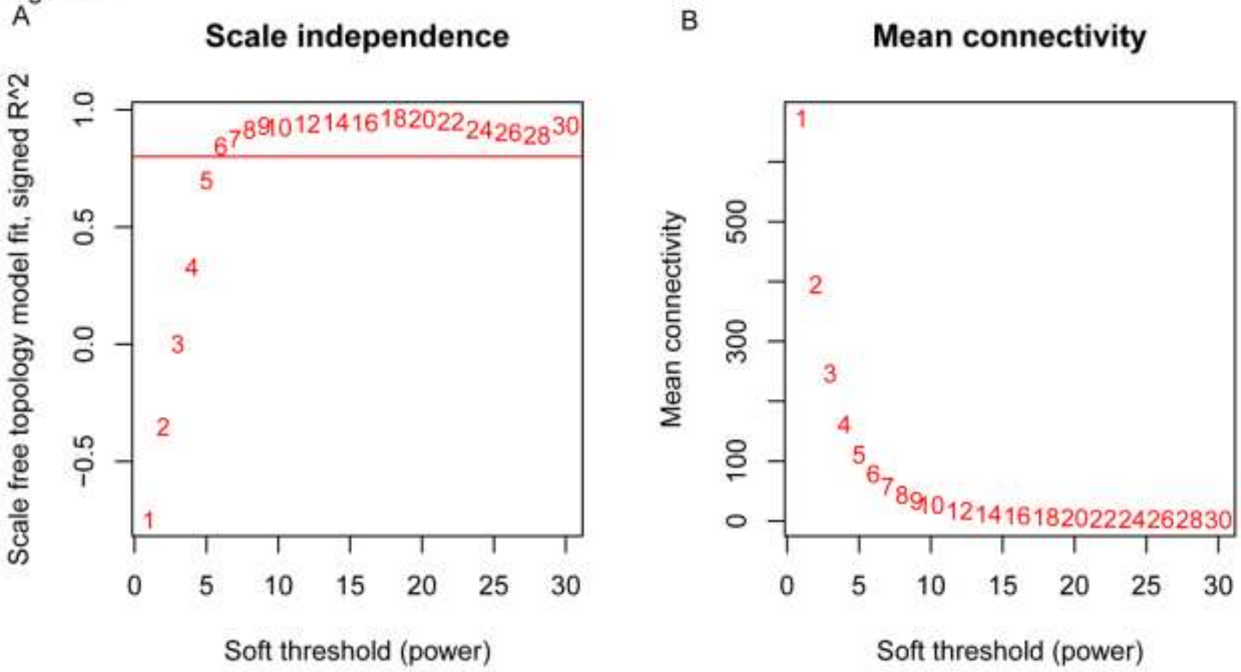

Figure 2S

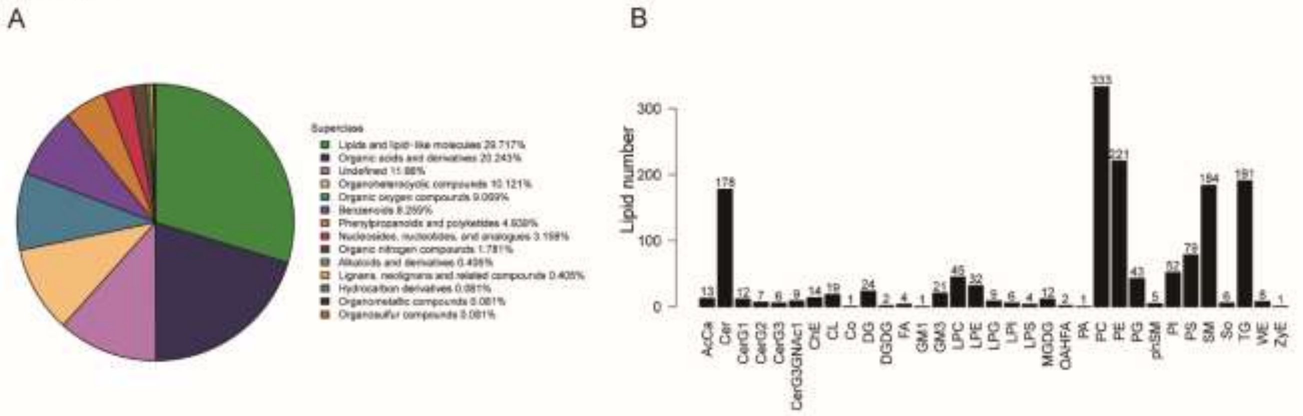

Figure S3

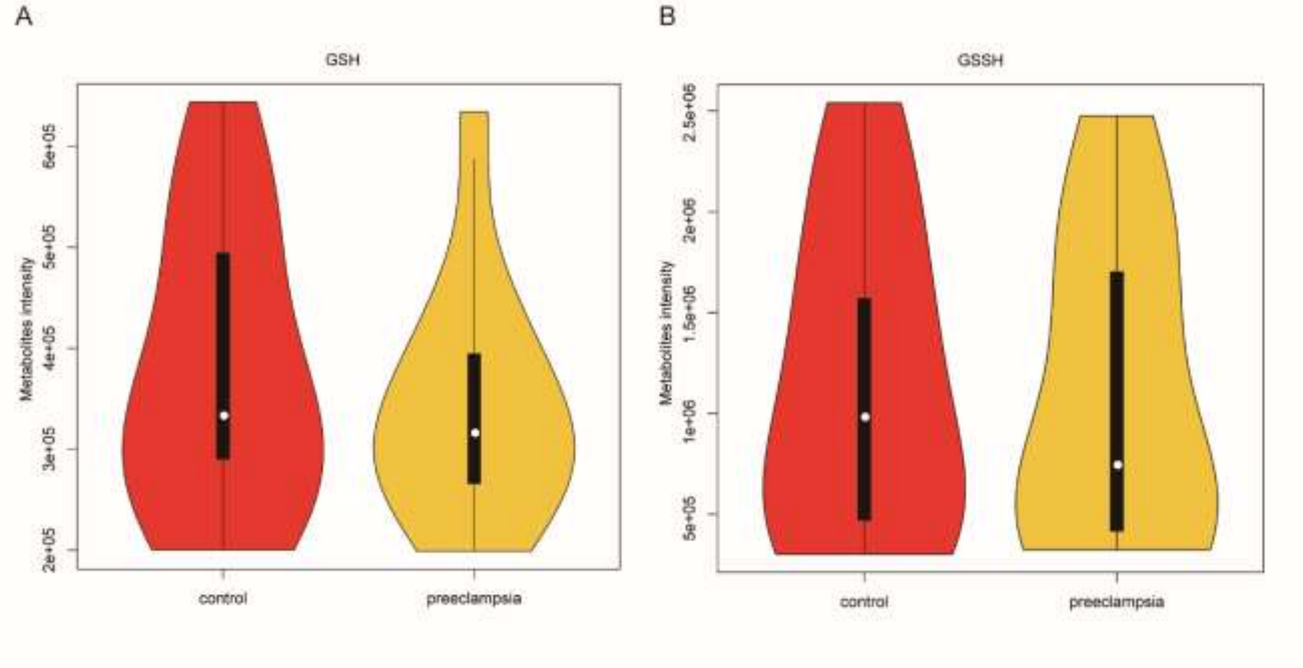

Figure S4

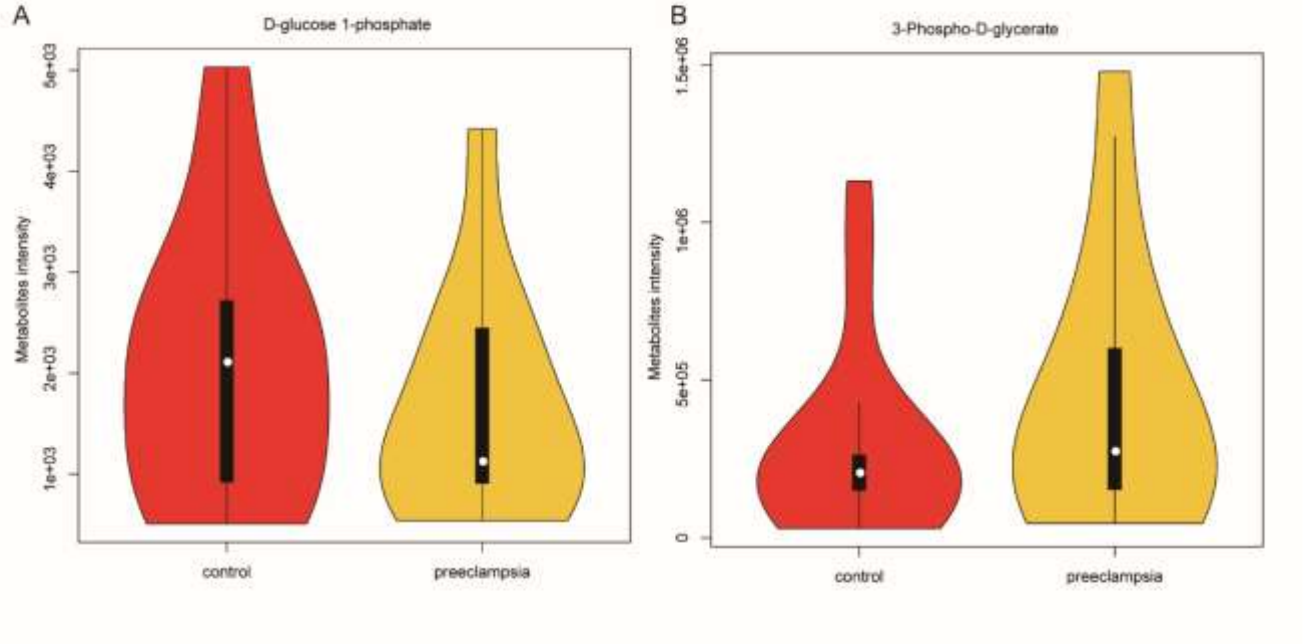

Figure S5

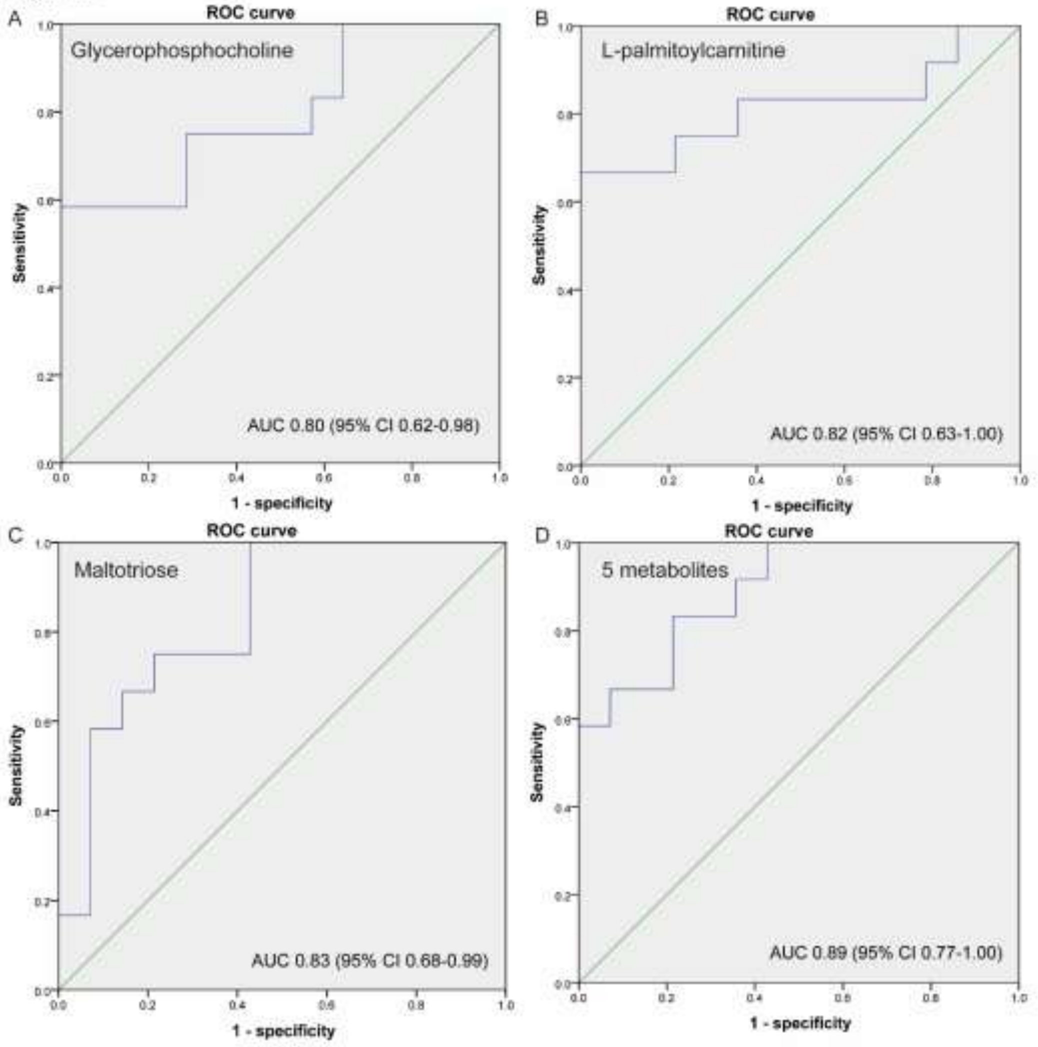

Figure S6

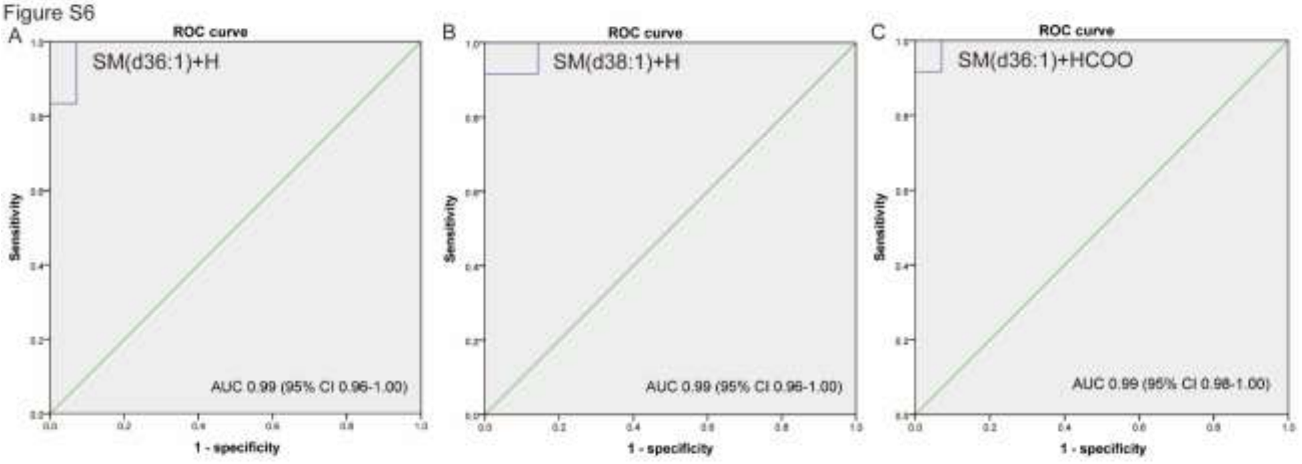

Supplement: Supplementary Figure 1 — Determination of soft-threshold power in the WGCNA. (A) Analysis of the scale-free index for various soft-threshold powers (β). (B) Analysis of the mean connectivity for various soft-threshold powers. [file Data_Sheet_1.PDF]
